# Down‐regulation of EOMES drives T‐cell exhaustion via abolishing EOMES‐mediated repression of inhibitory receptors of T cells in liver cancer

**DOI:** 10.1111/jcmm.15898

**Published:** 2020-12-16

**Authors:** Hongwei He, Yong Yi, Xiaoyan Cai, Jiaxing Wang, Xiaochun Ni, Yipeng Fu, Shuangjian Qiu

**Affiliations:** ^1^ General Surgery Jinshan Branch of Shanghai Sixth People's Hospital Shanghai Jiaotong University School of Medicine Shanghai China; ^2^ Key Laboratory for Carcinogenesis and Cancer Invasion Liver Cancer Institute, Zhongshan Hospital and Shanghai Medical School Fudan University Shanghai China; ^3^ General Surgery Shanghai Pudong Gongli Hospital Shanghai China; ^4^ Department of Anesthesiology Zhongshan Hospital Fudan University Shanghai China; ^5^ General Surgery Shanghai Ninth People's Hospital Shanghai China; ^6^ Department of Breast Surgery The Obstetrics & Gynecology Hospital of Fudan University Shanghai China

**Keywords:** eomesodermin, hepatocellular carcinoma, inhibitory receptors, PD‐1/CTAL‐4, T cells

## Abstract

T‐cell exhaustion is one of the hallmarks in cancer, but the mechanisms underlying T‐cell dysregulation remains unclear. Here, we reported that down‐regulation of transcription factor EOMES contributed to increased levels of inhibitory receptors in T cell among the tumour tissues and resulted in the poor prognosis of hepatocellular carcinoma (HCC). By analysing the correlation between EOMES in tumour‐infiltrating T cells and the clinical features, we demonstrated that the EOMES was related to the advanced stage and poor prognosis of HCC. Further mechanistic studies revealed that the EOMES mainly expressed in the CD8^+^ T cells and were down‐regulated in tumour samples. Moreover, we demonstrated that the EOMES directly bound at the transcriptional regulatory regions of the key inhibitory factors including PD‐1, CTAL‐4 and CD39, and lower levels of EOMES contributed to overexpression of these factors in T cells. Together, our studies provide new insight into the transcriptional deregulation of the inhibitory receptors on T cells during the tumorigenesis.

## 1 INTRODUCTION

Hepatocellular carcinoma (HCC) is a life‐threatening cancer, which causes about 700 000 death worldwide every year.^1^ Typically, the therapy of HCC includes surgery and chemotherapy, surgery at the early stages of the HCC resulted in a good outcome but the outcomes of most patients at the late stages of HCC remain unsatisfactory.^2^ Recently, immunotherapy targeting the microenvironment of tumour cells and its related immune cells has been applied in many malignant tumours including HCC. Some patients have achieved a successful response after therapy.^3^

Immune escape is one of the hallmarks of cancer.^4^ The immune cells lost the ability to clearance of the tumour cells or even promote the growth and invasion of tumour cells, such as the activation of the inhibitory between T cells and tumour cells.^3^ The cell surface receptors in T cells including PD‐1, CTAL‐4 and CD39 interact with the ligand expressed on the tumour cells and trigger a ‘do‐not‐eat me’ signal, which protected the tumour cells from T‐cell‐mediated clearance.^5^, ^6^ Targeting these immune blockades by using antibodies such as anti‐PD‐1 or anti‐PD‐L1 reactivated the effects of T cells on the tumour cells and achieved great successes in some patients with cancer including HCC.^7^, ^8^, ^9^ Still, many patients do not benefit from such therapy due to some unclear reasons. Thus, systematical unveiling the detailed mechanisms underlying the dysregulation of T cells in tumours is needed for developing new strategies of immunotherapy.

Eomesodermin (EOMES) is a T‐box transcription factor originally identified as a regulator of mesodermal development.^10^, ^11^ It initiates the transcriptional network governing the formation of endoderm via direct interaction with SMAD2/3 and transcription repressed the mesoderm genes.^12^ Lines of evidence also reveal that the EOMES regulates the maturation and function of CD8^+^ T cells,^13^ γδT cells^14^ and NK cells^15^ in response to pathogens. These studies focusing on the function of EOMES in T cells reveal a very important function of EOMES in response to pathogens. However, whether EOMES is dysregulated in tumour‐infiltrated T cells in HCC and the roles of EOMES in tumour‐infiltrating T cells remains largely unexplored.

Here, in our study, we analysed the expression of EMOES in the tumour‐infiltrated lymphocytes in 342 patients with HCC and found the high levels of EMOES expression were correlated with favourable prognosis of HCC. These observations were also supported by the TCGA cohorts. Further single‐cell transcriptomic analysis reveals the EMOES mainly expressed in the CD8^+^ T cells and dysregulated in tumour‐infiltrated CD8^+^ T cells as compared to the T cells in normal liver tissue. Moreover, ChIP‐seq re‐analysis revealed that the EMOES directly bound to the enhancer or promoter regions inhibitory receptors including PD‐1 (PDCD1), CD39 (ENTPD1) and CTLA‐4 (CTLA4).

## 2 MATERIALS AND METHODS

### 2.1 Patients and tissue specimens

A total of 342 patients with HCC underwent surgical resection at Zhongshan Hospital, School of Medicine, Fudan University, in 2007 and 2008 were enrolled in this study. This study was approved by the Research Ethics Committee of Zhongshan Hospital, School of Medicine, Fudan University. The inclusion criteria were as follows: (a) confirmed pathological diagnosis of HCC; (b) without preoperative anti‐cancer treatment or sign of distant metastasis; and (c) available clinicopathological characteristics and postoperative follow‐up data. The Barcelona Clinic Liver Cancer (BCLC) staging system was applied for clinical staging.^16^ Tumour differentiation was graded by the Edmond‐son‐Steiner grading system.^17^ Time to recurrence (TTR) and overall survival (OS) were defined as the interval from primary surgical resection to the first recurrence or death, respectively. The follow‐up was censored on 31 March, 2012.

### 2.2 Immunohistochemistry

Tissue microarray (TMA) was constructed as previously described.^18^ Triplicates of 1‐mm cores from two different areas (tumour centre and non‐tumour tissues that at least 1 cm away from tumour margin) were obtained for each case to ensure the reproducibility of the staining. The immunohistochemistry of the TMA chips was performed as previously described.^18^ Briefly, the TMA chip was deparaffinized in xylene and then hydrated with a graded alcohol series. The antigen retrieval was carried out in a pressure cooker (using a standard household model) with 0.1 mol/L EDTA (pH 8) for 9 minutes. The TMA chip was then placed in a blocking solution to inhibit endogenous peroxidase activity and non‐specific staining. The anti‐human eomesodermin (1:600; Abcam) was used for incubation at 4°C overnight. The TMA chip was incubated with the HRP‐Polymer anti‐Mouse/Rabbit antibodies (Maixin Bio Corp.) for 20 minutes at room temperature and stained with DAB for 3 minutes.

Blank controls were treated identically except the primary antibodies were omitted. The positive cells were observed with the use of a computerized image system composed of a camera connected to an Olympus U‐CAMD3 microscope (Olympus). Under high‐power magnification (×400), the number of eomesodermin‐positive cells (with brown granules in the nuclei^19^) in three visual fields in each 1‐mm cylinder was totally counted by two experienced pathologists who were blinded to the clinicopathological data of the patients. The assessments were performed independently, and disagreements were solved by discussion. Cell counts were expressed as the mean value of all nine fields.

### 2.3 Analysis of the single‐cell transcriptome data

The single‐cell transcriptome data of T cells isolated from patients with HCC or normal donors were retrieved from http://hcc.cancer‐pku.cn/.^20^ The expression of EOMES across the T cells, as well as the expression of EOMES in tumour‐infiltrating or normal T cells, was plotted according to the websites’ instructions.

### 2.4 ChIP‐seq re‐analysis

The ChIP‐seq of EOMES was retrieved from the Gene Expression Omnibus (GEO) database with the accession of GSM640691.^21^ The raw data were downloaded using the fastq‐dump algorithm. The fastq files were mapped to the hg38 reference genome using the bowtie2 software. The visualized files were generated by the homer suite.

### 2.5 Analysis of the expression of PD‐1, CTAL‐4 and CD39 in T cells with EOMES overexpression

The microarray data of the T cells with lentiviral transduction of EOMES were retrieved from the GEO database (GSE110545).^22^ The expression of the PD‐1, CTAL‐4 and CD39 was extracted using the R program under the ‘affy’ package.

### 2.6 Statistical analysis

Normally distributed continuous data were expressed as mean ± standard deviation (SD) and analysed using the Student's *t* test. Categorical data were presented as frequencies and analysed using the chi‐square test. Survival curves were computed using the Kaplan‐Meier method and analysed using the log‐rank test. All analyses were conducted using SPSS 17.0 (IBM, Armonk, NY, USA). Two‐tailed *P*‐values <.05 were considered statistically significant.

## 3 RESULTS

### 3.1 EOMES‐positive T cells were decreased in advanced stages of HCC

**Figure 1:**
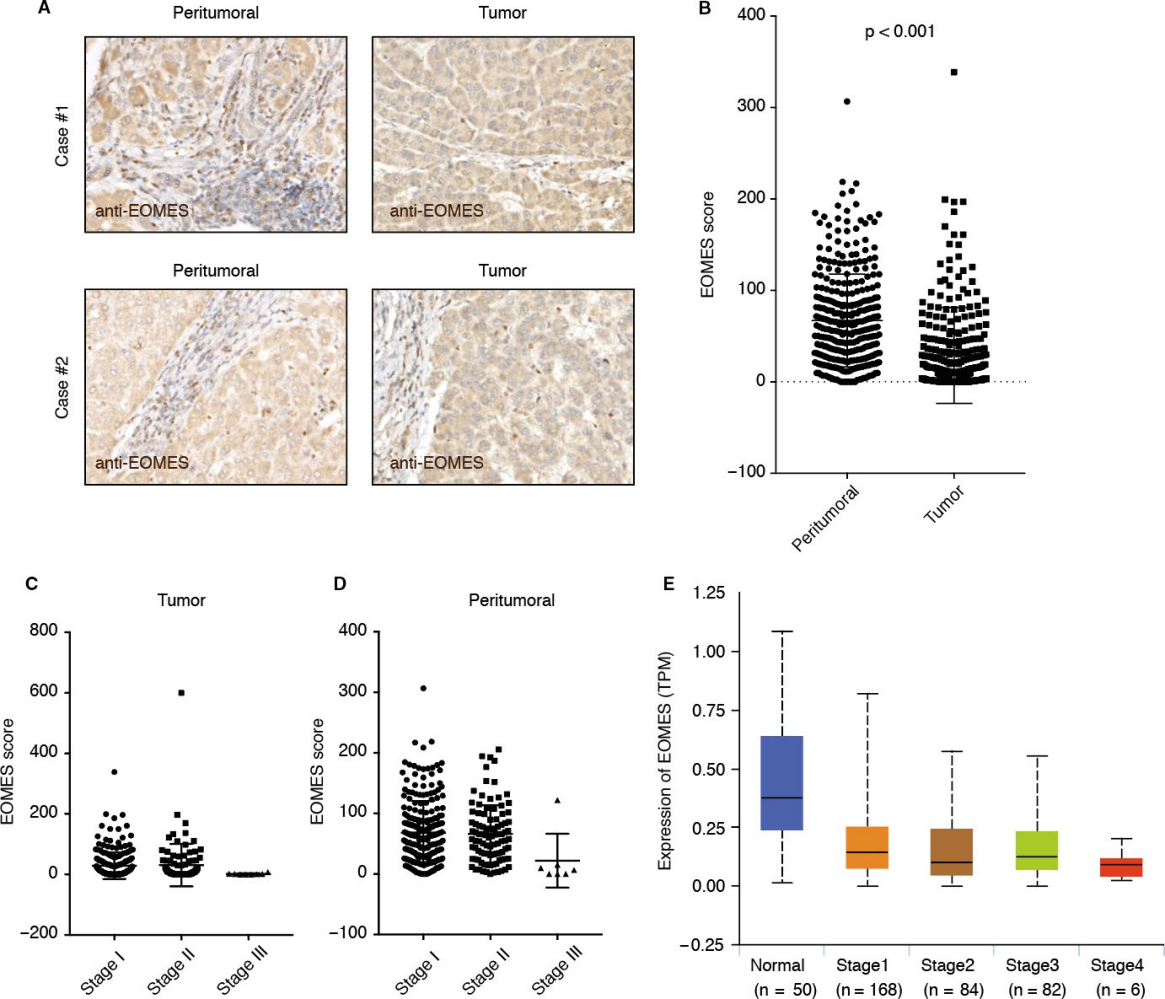
EOMES‐positive T cells were decreased in the tumour samples of patients with hepatocellular carcinoma (HCC). A, Representative immunohistochemistry for tumour and peritumoral eomesodermin protein expression in HCC. B, EOMES‐positive T cells were decreased in the tumour tissues of HCC as compared to the peritumoral sample. The EOMES score in tumour and peritumoral tissues of HCC were plotted. C, EOMES‐positive T cells were decreased in advanced stages of HCC. The EOMES score in tumour and peritumoral tissues in different stages of HCC were plotted. D, Validation using the TCGA cohorts. The mRNA expression of the EOMES in HCC tissues as well as the normal controls was plotted using the RNA‐seq data sets in the TCGA cohort

To investigate the role of EOMES in T cells, we first examined the expression of EOMES in tumour and peritumoral samples of HCC in a cohort containing 384 patients with HCC. The EOMES was mainly expressed in the nucleus of lymphocytes and the EOMES‐positive cells in peritumoral samples were significantly higher than those in tumour samples (Figure 1A‐B). Next, we determined the correlation between the EOMES‐positive score in tumour and peritumoral samples and the tumour stages of HCC. As shown in Figure 1C,D, the EOMES‐positive score was significantly decreased in advanced stages of HCC, but not significantly change in different stages of peritumoral samples. Also, we performed flow cytometry analysis in HCC samples and adjacent normal tissues and found the EOMES mainly expressed in the CD8‐positive T cells, and the EOMES levels in HCC samples were lower as compared to adjacent normal tissues (Figure S1).

Next, we collected the expression profiling of HCC samples in TCGA cohorts and determined the expression of EOMES in the RNA‐seq data of HCC samples as well as the normal control. In accordant with our observation using immunohistochemistry staining, the expression of EOMES was significantly decreased in tumour samples and stage IV HCC showed the lowest EOMES expression (Figure 1E).

We also determined the correlation between EOMES expression in tumour‐associated lymphocytes and the clinicopathologic characteristics of HCC patients. We found lower EOMES score was significantly enriched in tumour size >5 cm tumour tissues (*P* < .001) and stage III‐V patient samples (*P* = .005) (Table 1). But these were no significant correlation between EOMES score and gender, age, HBV infection, AFP score, cirrhosis and tumour number (Table 1). Suggesting down‐regulation of EOMES in the lymphocytes might contribute to HCC progression.

**Table 1:** Correlations between EOMES score and clinicopathologic variables

	EOMES score in HCC		*P* value
	EOMES High (n = 171, %)	EOMES Low (n = 169, %)	
Gender			
Male	146	142	.765
Female	25	27	
Age			
≤50 y	70	76	.511
>50 y	101	93	
AFP			
≤200 ng/mL	109	94	.151
>200 ng/mL	62	75	
Cirrhosis			
No	17	21	.495
Yes	154	148	
Tumour number			
Single	149	147	1.000
Multiple	22	22	
HBV infection			
No	52	41	.225
Yes	119	128	
Tumour size			
≤5 cm	134	93	<.001*
>5 cm	37	76	
Tumour differentiation			
Well and moderate	126	128	.709
Poor	45	41	
TNM stage			
I–II	90	63	.005*
III–IV	81	106	

Abbreviation: HCC, hepatocellular carcinoma. *p < 0.05.

### 3.2 Low levels of EOMES correlated with poor prognosis of HCC

**Figure 2:**
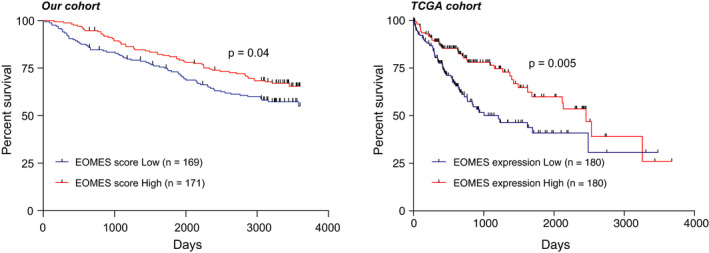
Lower levels of EOMES correlated with poor prognosis of hepatocellular carcinoma (HCC). A, Kaplan‐Meier curve of the overall survival based on the EOMES score in our cohort. The EOMES score was determined by IHC with antibodies against EOMES. Higher expression of EOMES in lymphocytes was divided as EOMES score high. Log‐rank analysis was used for statistical analysis. B, Kaplan‐Meier curve of the overall survival based on EOMES expression in HCC tissues in the TCGA cohort. The top 50% of EOMES expression was determined as EOMES high‐expression group, while the bottom 50% of EOMES expression was identified as EOMES low‐expression group. Log‐rank analysis was used for statistical analysis

To further investigate the prognostic value of EOMES in the tumour sample of HCC. We separate the HCC into two groups according to the EOMES score. We found that the lower EOMES score in tumour samples shown to be associated with poor prognosis of HCC (Figure 2A). Further analysis using the TCGA cohort further supported our finding (Figure 2B). Together, these observations suggested that the lower levels of EOMES‐positive cells might contribute to the development and progression of HCC and the expression of EOMES might serve as a marker for the prognosis of HCC.

### 3.3 The EOMES was decreased in tumour‐infiltrated CD8^+^ T cells

**Figure 3:**
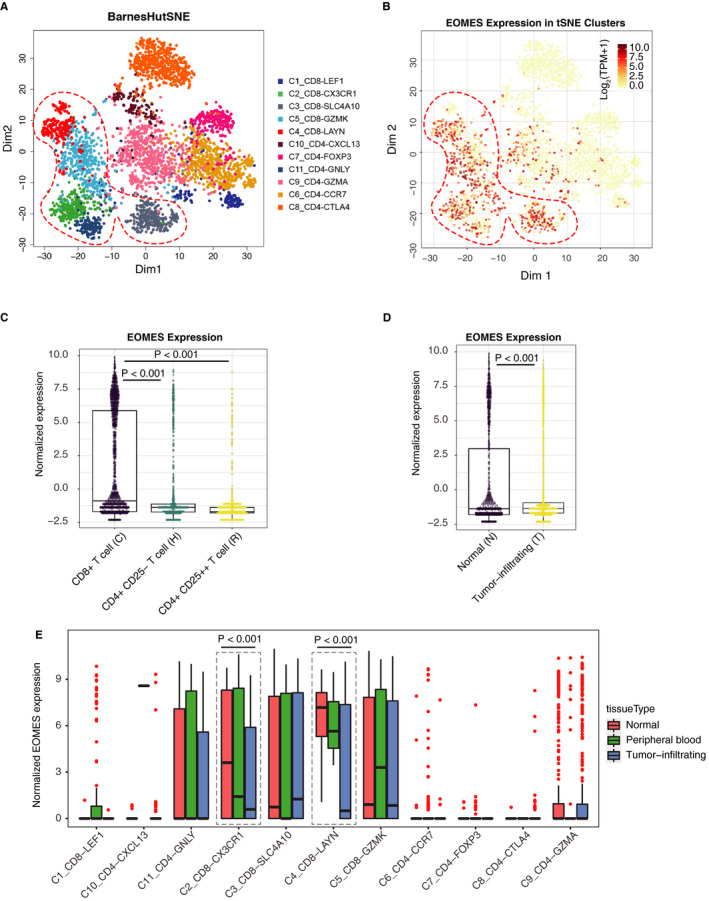
EOMES is down‐regulated in CD8‐positive T cells in tumour sample of hepatocellular carcinoma (HCC). A and B, t‐SNE clustering showing the expression distribution of EOMES across the different subtypes of T cells in HCC. C, EOMES mainly expressed in CD8‐positive T cells in HCC. D, The expression of EOMES was down‐regulated in tumour‐infiltrated T cells as compared to the T cells isolated from normal hepatocellular tissues. E, The EOMES was highly expressed in CD8_LAYN and CD8_CX3CR1 T cells. The expression of EOMES in different subset of T cells in normal liver tissues, peripheral blood and tumour samples was plotted

Prior studies using the EOMES score have demonstrated that the EOMES‐positive cells were decreased in advanced stages of HCC and lower levels of the EOMES‐positive cells shown to be a poor prognostic factors for HCC. However, the expression levels of EOMES in T cells remain obscure. We thus analysed the EOMES expression in distinct subtypes of T cells in the single‐cell transcriptomic analysis of T cells in HCC. We found that the EOMES mainly expressed in CD8^+^ T cells (Figure 3A‐C). We next compared the expression of EOMES in HCC tumour samples and normal hepatocellular tissues, we found that the expression of EOMES in normal hepatocellular tissues was much higher than those in HCC tumour samples (Figure 3D), indicating that in addition to decreased EOMES‐positive cells, the expression of EOMES was also down‐regulated in the T cells of patients with HCC. Next, we analysed the EOMES gene expression within the same clusters of T cells and found the CX3CR1‐CD8 T cells and LAYN‐CD8 T cells shown the highest EOMES expression. The EOMES in these two subsets of T cells was significantly higher in normal liver tissue than those in tumours.

To further support the correlation between CD8 T cells and EOMES expression, we used two deconvolution tools, including CIBERSORTx^23^ and Xcell,^24^ to compute the infiltration of immune cells in the microenvironment of liver cancer tissues. The enrichment score of infiltrated B cells, T cells, NK cells and other immune cells was obtained using the RNA‐seq data sets of TGCA‐LIHC. We then calculated the correlation between enrichment score of each immune cell populations and the expression levels of EOMES. We found the expression of EOMES was positively correlated with CD8 T cells (Figure S2), suggesting that the EOMES might be expressed in CD8 T cells.

### 3.4 EOMES regulates the expression of inhibitory receptors of immune checkpoints

**Figure 4:**
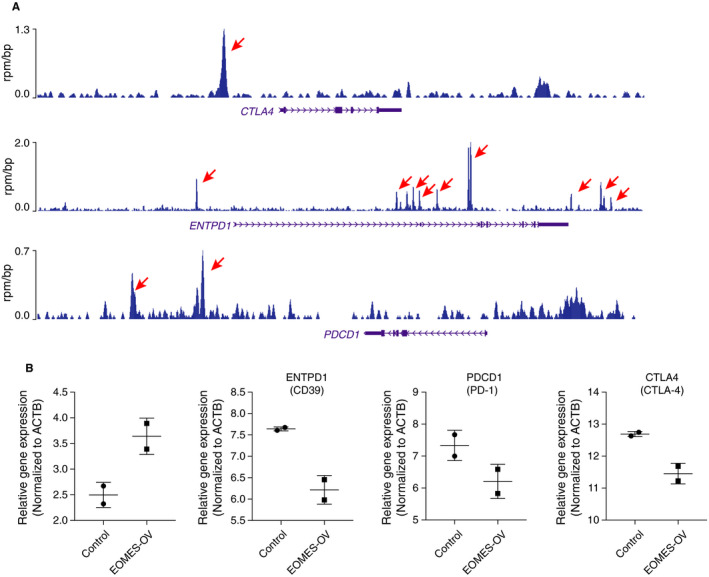
A, EOMES repressed the expression of inhibitory receptors in T cells. EOMES directly bound at the transcriptional regulatory regions of PD‐1 (PDCD1), CTAL‐4 (CTLA4) and CD39 (ENTPD1). B, EOMES overexpression decreased the expression of PD‐1 (PDCD1), CTAL‐4 (CTLA4) and CD39 (ENTPD1) in T cells

Since EOMES functions as a transcription factor in gene regulation, we speculate that the deregulated EOMES might also contribute to the deregulation of genes important in T cells of HCC. We thus retrieved the ChIP‐seq data of EOMES in the GEO database (GSM640691). Interestingly, we found that the EOMES directly bound to many genes involved in T cells regulation (Figure 4A). We found EOMES directly bound to the regulatory region of PD‐1 (PDCD1), CD39 (ENTPD1) and CTLA‐4 (CTLA4) (Figure 4A). These factors are key inhibitory factors of immune checkpoints, suggesting a potential role of EOMES in the regulation of immune checkpoints of T cells.

We next retrieved a gene expression data set that contains the vector and EOMES overexpression lentiviral‐transduced T cells. We compared the expression of EOMES, PDCD1, ENTPD1 and CTLA4 in the vector (Control) or EOMES‐overexpressed (EOMES‐OV) T cells. We found that the EOMES overexpression leads to the down‐regulation of PDCD1, ENTPD1 and CTLA4 expression (Figure 4B), indicating high levels of EOMES might contribute to decreased expression of the inhibitory receptors.

Together, the ChIP‐seq and EOMES overexpression results suggesting that the EOMES might directly bound and repressed the expression of inhibitory receptors, and this repression might benefit the T‐cell‐mediated immune response on tumour cells. However, the EOMES was down‐regulated in HCC which might partially explain the exhausted T‐cell response in HCC.

## 4 DISCUSSION

T‐cell exhaustion is one of the hallmarks of cancer, but the mechanisms underlying T‐cell dysfunction remain unclear. In this study, we reported that the down‐regulation of EMOES in tumour‐infiltrated T cells and the decrease of the EMOES‐positive T cells in tumours were poor prognosis factors for hepatocellular carcinoma.

Emerging evidence demonstrated T‐cell exhaustion is one of the hallmarks of malignant diseases. The tumour cells can escape from the killing of T cells by activation of PD‐1/PD‐L1, CTLA4/CD80/CD86 and other immune checkpoints. The PD‐L1 gene in tumour cells is shown to be activated in many malignancies while the PD‐1 gene is also reported to be activated in tumour‐infiltrated T cells. The mechanisms underlying aberrant PD‐L1 expression have been intensively studied, but the mechanism underlying high level of immune checkpoint protein, such as PD‐1 and CTLA4, in tumour‐infiltrated T cells remain largely unexplored. We here reported that the EOMES gene was down‐regulated and contributed to highly expressed PD‐1 and CTLA4 in tumour‐infiltrated T cells. Noteworthy, several studies also reported that the higher levels of PD‐1 in T cells are a poor prognostic factors for patients without immune therapy in many cancers including liver cancer.^25^, ^26^, ^27^, ^28^ We here reported the lower levels of EOMES might contribute to the higher levels of PD‐1 and resulted in an unfavourable prognosis of liver cancer.

EOMES is a transcription factor mainly expressed in T cells and NK cells. Normally, EOMES enhances the expression of IFNγ in CD8^+^ T cells.^13^, ^29^, ^30^ In addition, the EOMES expression is increased when T cells adopt a memory‐like phenotype.^31^, ^32^, ^33^, ^34^ The EOMES‐high cells produce fewer cytokines and higher levels of granzyme‐B and show higher expression of PD‐1 than T‐bet‐high cells during chronic infection.^13^, ^35^, ^36^, ^37^ Ectopic expression of EOMES in murine cells led to enhanced expression of perforin and granzyme‐B,^36^ indicating the eomesodermin is responsible for cytolytic functions. Also, several studies also demonstrated that the Eomes down‐regulated the expression of PD‐1 in mice,^38^ which is similar to our findings that the EOMES might down‐regulated the transcription of PD‐1 through direct transcriptional regulation. In addition, we also found that EOMES might also down‐regulated the expression of CTLA4 and CD39 via direct transcriptional regulation, and more detailed mechanisms are needed to be explored in the future.

EOMES has been reported to control the type of adaptive immune responses within the tumour site via promoting Th1 fate and suppressing Th17 differentiation.^13^, ^39^ It suppresses the promoter activity of Rorc and IL‐17a through directly transcriptional regulation.^40^ We showed that EOMES expression was higher in peritumoral liver tissues than in HCC tissues. The lower expression levels of EOMES in tumour‐infiltrated T cells were associated with advanced stages and poor prognosis of HCC. EOMES has been suggested to play an important part in controlling CD8^+^ T‐cell activity and lymph node metastasis in human colorectal cancer.^37^ Dielmann et al^41^ showed that high EOMES expression was associated with better survival of patients with renal cell cancer treated with sorafenib, suggesting that lower levels of EOMES might be a common signature for cancer with poor prognosis. Still, additional studies are necessary to examine the exact mechanisms involved in these observations.

## CONFLICT OF INTEREST

All authors declare that they have no competing interests.

## AUTHOR CONTRIBUTIONS

**Hongwei He:** Conceptualization (equal); Data curation (equal); Formal analysis (equal); Funding acquisition (equal); Investigation (equal); Methodology (equal); Project administration (equal); Resources (equal); Software (equal); Supervision (equal); Validation (equal); Visualization (equal); Writing‐original draft (equal); Writing‐review & editing (equal). **Yong Yi:** Data curation (equal); Formal analysis (equal); Funding acquisition (equal); Investigation (equal); Methodology (equal); Project administration (equal); Resources (equal); Software (equal); Validation (equal); Visualization (equal); Writing‐review & editing (equal). **Xiaoyan Cai:** Data curation (supporting); Investigation (supporting); Resources (supporting); Software (supporting); Validation (supporting); Visualization (supporting). **Jiaxing Wang:** Data curation (supporting); Formal analysis (supporting); Investigation (supporting); Methodology (supporting); Validation (supporting). **Xiaochun Ni:** Data curation (supporting); Investigation (supporting); Methodology (supporting); Project administration (supporting); Validation (supporting); Visualization (supporting). **Yipeng Fu:** Investigation (supporting); Project administration (supporting); Validation (supporting). **Shuangjian Qiu:** Conceptualization (lead); Funding acquisition (lead); Supervision (lead); Writing‐original draft (lead); Writing‐review & editing (lead).

## Supporting information

Figure S1‐S2

## Data Availability

The data that support the findings of this study are available from the corresponding author upon reasonable request.
