# Supplementary material for: Down‐regulation of EOMES drives T‐cell exhaustion via abolishing EOMES‐mediated repression of inhibitory receptors of T cells in liver cancer
Source: J Cell Mol Med. 2020 Dec 16;25(1):161–9. doi: 10.1111/jcmm.15898 (PMC7810931; doi:10.1111/jcmm.15898)
Supplement: Supplementary file 1 — Figure S1‐S2 [file JCMM-25-161-s001.docx]

**Supplementary Figures**


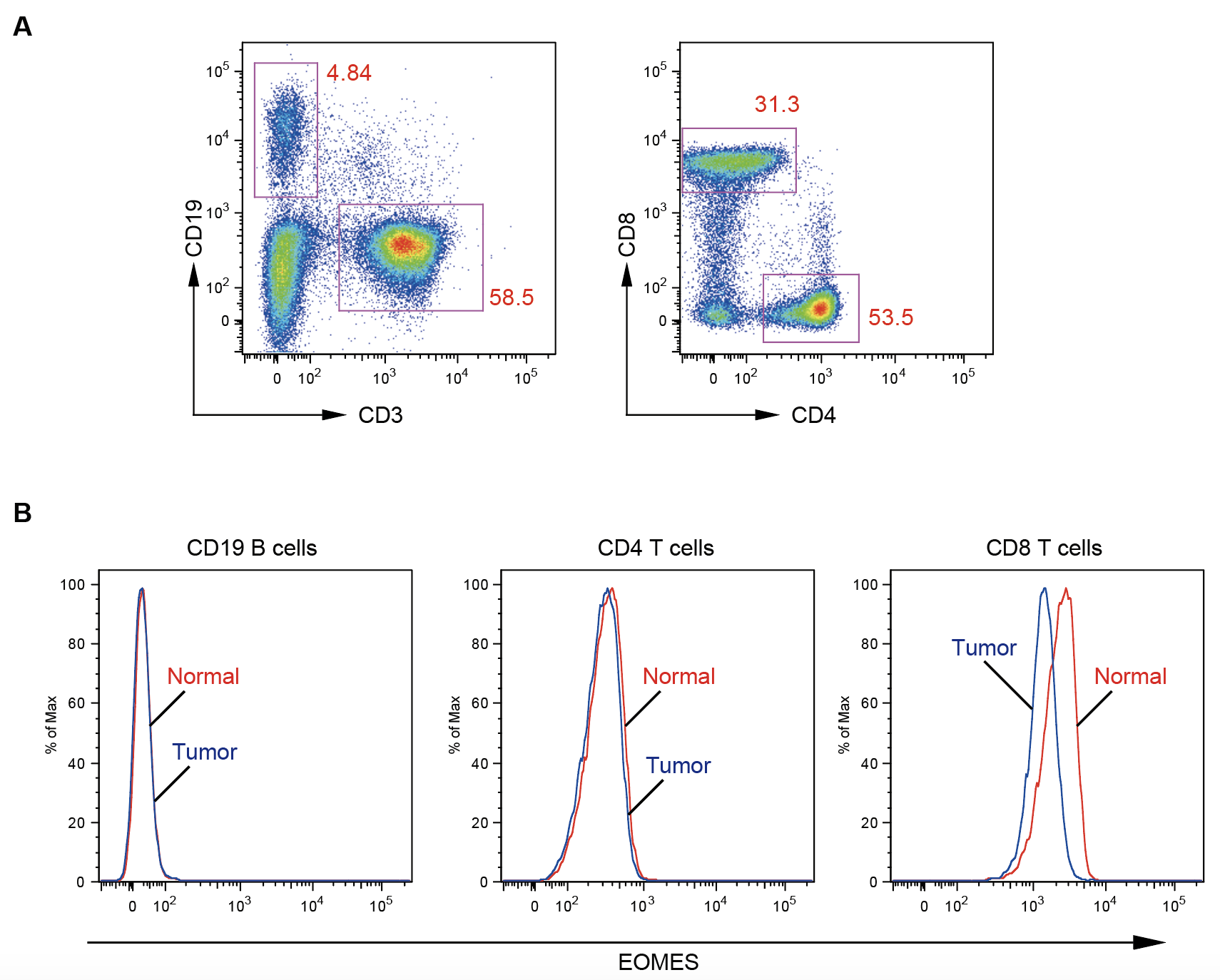


**Figure S1. EOMES mainly expressed in CD8^+^ T cells.** (A) Isolation of immune cells. The representative distribution of isolated immune cells was shown. (B) The expression of EOMES in CD4^+^, CD8^+^, and CD19^+^ cells in liver cancer and the adjacent normal tissues were shown. The liver cancer tissue and the adjacent normal tissues were digested and stained with antibodies against CD3, CD4, CD8, CD19, CD56 and EOMES. The expression of EOMES in CD4^+^, CD8^+^, and CD19^+^ cells were determined. No CD56 positive cells (NK cells) were observed.


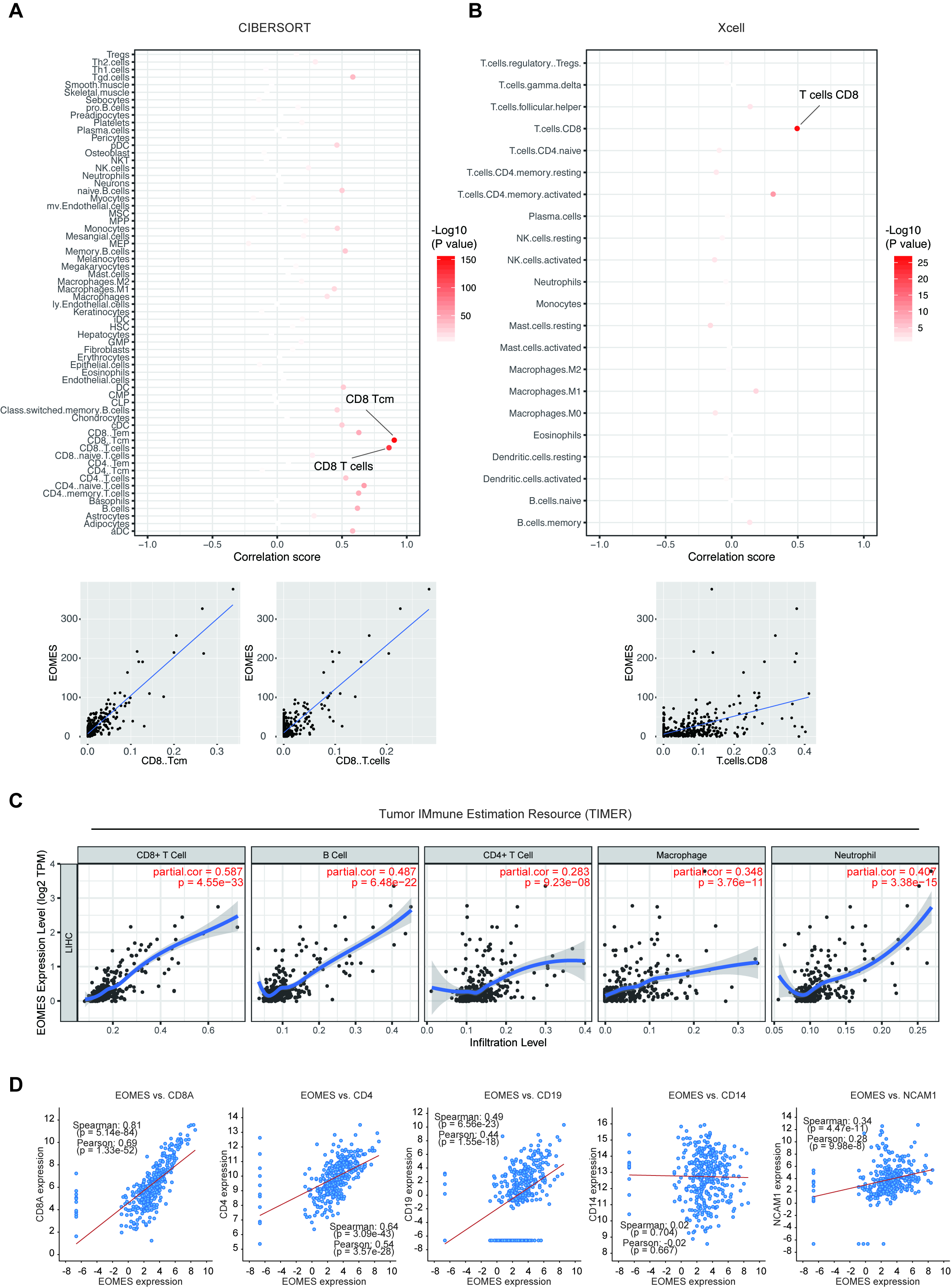


**Figure S2. EOMES were highly correlated with CD8 T cells.**

(**A**-**B**) Correlation between EOMES expression and the infiltration score of the immune cells. The infiltration scores were calculated by the CIBERSORTx (A) and Xcell (B) using the RNA-seq data in TCGA-LIHC. Correlation score and relative p value were plotted in the upper panels. Correlation between EOMES and CD8 infiltration scores were plotted in the lower panels. (**C**) The correlation of the EOMES expression with immune infiltration level in LIHC. The plots were generated with the online tools of the Tumor IMmune Estimation Resource (TIMER). (**D**) Correlation between the expression of EOMES and CD8, CD4, CD19, CD14 and NCAM1 (CD56). The expression data of these genes were obtained from the TCGA-LIHC RNA-seq datasets.
